# Trends in Population-Based Studies: Molecular and Digital Epidemiology (Review)

**DOI:** 10.17691/stm2022.14.4.07

**Published:** 2022-07-29

**Authors:** N.S. Denisov, E.M. Kamenskikh, O.S. Fedorova

**Affiliations:** Assistant, Research and Educational Laboratory “Live Laboratory of Population-Based Studies”; Siberian State Medical University, 2 Moskovsky Trakt, Tomsk, 634050, Russia; Head of the Research and Educational Laboratory “Live Laboratory of Population-Based Studies”; Siberian State Medical University, 2 Moskovsky Trakt, Tomsk, 634050, Russia; Professor, Head of Department of Faculty Pediatrics with the Course of Childhood Diseases, Faculty of General Medicine; Siberian State Medical University, 2 Moskovsky Trakt, Tomsk, 634050, Russia

**Keywords:** population-based studies in medicine, digital epidemiology, molecular epidemiology, omics technologies, information technologies

## Abstract

The development of high-throughput technologies has sharply increased the opportunities to research the human body at the molecular, cellular, and organismal levels in the last decade. Rapid progress in biotechnology has caused a paradigm shift in population-based studies. Advances in modern biomedical sciences, including genomic, genome-wide, post-genomic research and bioinformatics, have contributed to the emergence of molecular epidemiology focused on the study of the personalized molecular mechanism of disease development and its extrapolation to the population level. The work of research teams at the intersection of information technology and medicine has become the basis for highlighting digital epidemiology, the important tools of which are machine learning, the ability to work with real world data, and accumulated big data.

The developed approaches accelerate the process of collecting and processing biomedical data, testing new scientific hypotheses. However, new methods are still in their infancy, they require testing of application under various conditions, as well as standardization. This review highlights the role of omics and digital technologies in population-based studies.

## Introduction

The global medical and demographic problems, those of population ageing, an increase in the prevalence of chronic non-communicable diseases, the pandemic of a new coronavirus infection, set new large-scale challenges for healthcare, where precision medicine becomes one of the tools for solving them. Initially demanded mainly in the diagnosis and treatment of oncological diseases, it is being introduced into all areas of medicine now.

Major research projects and campaigns are being initiated worldwide to develop and implement precision medicine strategies. Experts estimate the global precision medicine market to reach $87.7 billion by 2023. The leading scientific institutions are located in the USA, United Kingdom, France, and China. Since 2018, the number of publications in the field of precision medicine has amounted to about 16 thousand worldwide.

Molecular and digital epidemiology is one of the main tools of precision medicine.

## Molecular epidemiology

### Genomic research

Biological research has traditionally been carried out using reductionist approaches, partly due to limitations in both the experimental power of the devices and the complexity of the analytical data evaluation processes. In the last decade, the development of high-throughput technologies has led to a sharp increase in the opportunities of studying the human body at the molecular, cellular, and organismal levels [1]. The rapid progress of biotechnology has led to a paradigm shift in genomic epidemiology, from linkage analysis to genome-wide association studies (GWAS) and the widespread use of next-generation sequencing (NGS). Technological developments have improved research design, enhanced our understanding of disease etiology, and led to numerous scientific discoveries [2].

In genomics, first-generation sequencing methods could sequence the human genome for $300,000; two decades later, next-generation methods can sequence the human genome in a few hours at a cost of $1000. Measurements of characteristics such as epigenome, transcriptome, proteome, etc. have undergone similar changes, which has allowed researchers to start studying pathologies using their characteristics at the molecular level rather than tissue one [3]. Therefore, both the study of individual organisms and the study of populations require calculative and statistical approaches to the data of various “omics”, which consider metabolism in cells, tissues and organs as a whole, as an integrated system, rather than isolated separate processes.

The reductions in the cost of genome sequencing, combined with an increase in the computational power, have caused a strong revival of interest in the application of whole genome sequencing in public health [4]. Today, genomic epidemiology makes it possible to study the genomes of pathogens so as to have a better insight into the spread of infectious diseases among populations and quickly respond to the outbreaks of the diseases [5]. Together with philodynamics (a combination of epidemiology, evolution, and immunodynamics), genomic epidemiology is a rapidly developing field of science that addresses key issues related to epidemic preparedness and management in real time [6].

In the beginning, genomic data were used to study a variety of viruses, particularly, the influenza A virus and human immunodeficiency virus (HIV). The Ebola virus epidemic in West Africa (2013–2016) was the first major and large-scale challenge to study the virus genomes; that resulted in the discovery of their origin and causes for such a rapid spread of the epidemic and also allowed to detect subsequent sources of local outbreaks [7].

Genomic epidemiology has become a valuable source of information for scientists about the nature of the threats to public health such as Zika, Middle East Respiratory Syndrome (MERS), Ebola, and SARS-CoV-2 outbreaks [8]. These threats have required a variety of approaches including intensive genome sequencing to understand transmission dynamics during the acute phase of epidemics (Ebola virus in the Democratic Republic of the Congo) and broader genomic “surveillance” to detect a hidden increase in the prevalence (poliomyelitis) [9]. During the SARS-CoV-2 pandemic, many countries that had not previously used genomic data began to actively conduct such studies and rely on their results. Genomic technologies have made more than 2.5 million SARS-CoV-2 sequences known from over 185 countries [10], and due to the subsequent public interest in genomic epidemiology, new methodologies have been rapidly developed to fully utilize this dataset to fight against the pandemic.

The transmission of all infections occurs at different spatial scales, which depend on the pathogen, the nature of the host’s movement, immunity, and other factors [11].

**Figure 1.**
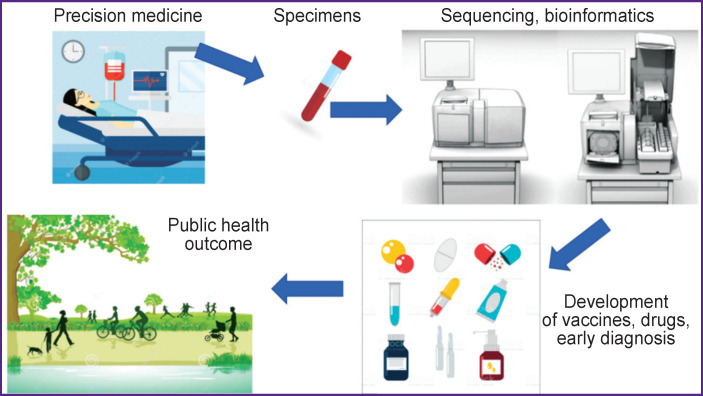
Pathogen sequencing during infectious disease outbreaks Schematically presented relationship between obtaining genomic data and creating health-saving technologies

The impact of obtaining genomic data on the formation of public health is shown in Figure 1.

Genomic data can be used to characterize clinical cases of infection depending on location and time and track outbreaks at all spatial scales: from nosocomial infections to pandemics [12]. The analysis of the pathogen genomes in the context of other sequences obtained from the same outbreak, as well as their comparison with previously characterized variants, allow researchers to develop intervention strategies at the individual and population levels to minimize the burden of infectious diseases on the individual and society [13]. This comprehensive approach involving pathogen sequencing, analysis, and response is called molecular epidemiology. In contrast to the development of individual-level treatment strategies that focus on the functional roles of host and/or pathogen mutations, the outbreak-scale genomic analysis uses pathogen mutations as markers of transmission events [14].

Genomic epidemiology studies the dynamics of outbreaks and the rapid evolution of pathogens that often accumulate mutations on the same scale as the spread of these pathogens.

NGS makes it possible to detect various types of genomic and epigenetic variations with high accuracy. Such sequencing allows researchers to directly study all these variations in person, increasing the chance of detecting mutations [15]. Although the use of NGS is still limited due to its high cost, the success of several recent projects demonstrates the great potential of this method in genomic epidemiology, especially in view of the sequencing cost decline.

With a sufficient sample size, appropriate metadata (such as location and date), and an appropriate statistical framework, pathogen genomes may assist in the identification of patterns in the spread of an epidemic with a small number of patients studied, allowing the development of precise targeted interventions compared to traditional methods and the use of demographic data [16]. In the nearest future, we will also be able to estimate the prevalence of chronic noncommunicable diseases using patient’s pedigree data.

In 2011, the National Human Genome Research Institute (USA) published a review on genetic medicine, noting that the most effective way to improve human health is to understand normal biology (in this case, biology of the human genome) as a basis for studying the biology of diseases, which then becomes the basis for health promotion. To date, it is still difficult to fully determine the future prospects of genetic epidemiology for improving the public health [17].

When evaluating the contribution of genetic epidemiology to public health, it is equally important to understand that the etiology of diseases is complex and the genetic risk for developing pathology does not equate to genetic determinism [18]. The complex relationship between genetics and disease poses an ethical dilemma for practitioners regarding the correct interpretation of genetic test results. When performing genetic tests, it is possible to indirectly reveal the disorders that will not cause the development of the clinical disease manifestation [19]. An ethical question arises, should patients be aware of these incidental findings that may have a medical value?

### Biomarkers

In the epidemiological study of diseases, metabolite concentrations are increasingly used as biomarkers that serve as indirect indicators of the rate of metabolic reactions. Though, the assessment of the rate of individual reactions can provide more accurate information about the ongoing changes directly in the organ [20].

Direct measurement of the rate of metabolic reactions *in situ* is currently impractical in large population studies since they are costly, technically complex, and require high-throughput equipment. This method is more successful when applied on a smaller scale, primarily through the use of non-invasive nuclear magnetic resonance spectroscopy (NMR spectroscopy) [21]. Metabolic pathway imaging techniques using hyperpolarized metabolites have shown promising results in the diagnosis and localization of tumors in patients with prostate cancer [22].

In a prospective clinical study involving 58 patients with chronic heart failure, the rate of adenosine triphosphate (ATP) synthesis was measured by studying the activity of cardiac creatine kinase *in situ* using the 31P NMR spectroscopy method [23].

ATP and creatine phosphate concentrations, as well as general clinical parameters, were used as predictors of chronic heart failure over an 8-year follow-up period. Excessive creatine kinase activity exceeded the significance of such parameters like patient’s age, gender, and concentrations of other metabolites in predicting heart failure events and death, including hospitalization for heart failure and ventricular assist device insertion [24].

These results relate to a relatively small group of patients, but they add weight to the case for the development of biomarkers based on the rate of metabolic pathways and reactions in the study of disease.

Metabolism works as a continuously operating system of movement and transformation of molecules through reactions. Since the flow of metabolites is regularly redirected, metabolites are accumulated at various points or become depleted which results in a change in their concentration. The concentrations of metabolites reflect the effects of combined changes in the reaction rate, but do not give a direct idea of the dysfunctions of the processes themselves, for example, in pathology affecting enzymes, genes, and other molecular products derived from the human genome [24]. In this regard, a systematic assessment of the reaction rate on the scale required for epidemiology will be done by integrating metabolomic data with genomic, transcriptomic and/or proteomic information to determine enzymatic function.

Due to the ability to characterize diverse variants of endogenous and exogenous metabolites in biological specimens, metabolomic approaches have quickly been recognized as an important tool in public health studies [25]. The results show that the use of small volumes of blood, urine, feces, saliva, exhaled air condensate, cerebrospinal fluid, and biopsy for measuring the metabolome can provide information on possible mechanisms underlying the disease [26-30]. However, most of the existing evidence has come from case–control or crossover studies, which do not allow for a clear temporal relationship between exposure, biomarkers, and disease.

Recently, the metabolic characterization of amniotic fluid, cord blood, and maternal/child urine or serum samples has been used to assess complex effects on the fetus and mother, and it may potentially be associated with developmental problems. Dried newborn blood spot used to identify metabolic biomarkers of future risk for cancer and other diseases have been proposed as a promising sample for metabolomic profiling [31-34].

The application of metabolomics for the study of disease risks, screening, and treatment efficacy has yielded promising initial results, although the field is still under development. These studies include ones on neurodegenerative diseases [35], type 2 diabetes [36], cancer [37], HIV, tuberculosis [38], malaria [39], and cardiovascular diseases [40]. The next important step in the application of metabolomics to study the etiology of diseases and early detection of pathologies will be longitudinal studies, which have already shown their effectiveness in creating biological models of the environmental impact on humans [41, 42].

## Digital epidemiology

To conduct large multicenter epidemiological studies, digital technologies are actively used to facilitate the processes of work planning, data remote collection and entry control, as well as subsequent result presentation and reuse [43-46].

Though the epidemiology of chronic noncommunicable diseases in Russia is still lagging behind infectious diseases [47], there is a need to create and implement digital services for epidemiology of chronic noncommunicable diseases [48]. The need is owing to the increase of omics technologies’ availability, the accumulation of the many years results of research, the need to compare the findings of similar studies, and the increased requirements for practical application and implementation of the results [47, 49].

### Digital systems for clinical research

The basis for conducting research in the field of precision medicine is the formation of databases of clinical information annotated with the data on the collected biomaterials for each clinical case [50, 51]. This significantly expands resource opportunities for research at the intersection of clinical areas when new members of the research team are involved or in the case of a long-term work [52].

Coppola et al. [50] emphasized the importance of combining primary data with paraclinical information, including data from imaging studies, in a digital system. According to the authors, a service for visual data processing should have the options not only to display, but also to analyze data, which requires pre-processing and data markup. The selection of areas with suspected lung infiltration according to computed tomography (CT) data or with pathological signal foci in the magnetic resonance imaging (MRI) pictures can be an example. Integration of genomic analysis into the data system contributes to the development of genomics and radiomics (radiomics is aimed at creating mathematical models and computer algorithms that, through the analysis of medical images, such as MRI or CT images, provide a finding about the pathophysiological features of tissues) [50, 53]. According to the research teams accumulating biomedical data, the imaging biobank data are to be used in accordance with the already-known standards until specific standards have been developed [50, 54]. Harmonization of processing will make it possible to combine data from multi-omics studies and visual materials for the integration of phenotypic and genotypic data [50, 55].

Over the past 10 years, many medical institutions have collected integrated databases (integrated data repositories, IDRs) [56], which are collected from electronic medical records [57]. Based on the accumulated data, not only scientific hypotheses are tested, but also a clinical decision support system is built [56]. Gagalova et al. [56] identified four models for the architecture of medical data collection and storage, in which data sources, the purpose of use, the availability of storage, etc. The purpose of this work was to initiate the development of guidelines on IDR creation in hospitals.

### Online databases

Interactive monitoring systems have gained wide popularity [58]. Over the past 20 years, many services for monitoring infectious diseases have emerged [59, 60]. To monitor the situation with antibiotic resistance, many services have been created that are limited geographically as well as by described microorganisms and assessed metrics:

EARS-Net (https://atlas.ecdc.europa.eu/public/index.aspx);

CDDEP Resistance Map (https://resistancemap.cddep.org/index.php);

SGSS (https://sgss.phe.org.uk/Security/Register);

ATLAS (https://atlas-surveillance.com/#/login);

SMART (https://globalsmartsite.com/#/auth/login).

The free-access web application AMRmap (https:// amrmap.ru/) [61] is a Russian development which displays data on antibiotic resistance obtained in multicenter clinical trials. The system has a section of genetic markers. Information in the database has been stored since 1997, access provided free of charge.

Since 2018, the University of Bristol’s project EpiGraphDB [62] has been developing, which is a data-based analytical platform designed for the intellectual analysis of epidemiological indicators. The project is developing approaches to the interpretation of causal relationships in the systematic automated analysis of many phenotypes using data from the array of bioinformatic resources. The university is also developing a software for statistical processing of omics studies, MR-Base being an example of it [63].

A large system of producing sequences of biological reactions in the body is presented in the WikiPathways system [64]. Currently, this system is being actively filled out with omics research data. The STRING database contains known and predicted protein–protein interactions [65].

Toom et al. [66] compared the results of an epidemiological study of headache in Estonia using an online questionnaire with the results of data research obtained during face-to-face visits of patients. The use of online questionnaires can significantly speed up the data collection process, increase population coverage, and reduce manual data entry errors. However, the authors noted that in the online survey, the majority of people did not have a headache, which greatly differed the sample of people who completed the online questionnaires from the sample of patients who came for face-to-face visits. This reduced the incidence of headache in the population. Also, more women, young people, married people, urban residents and people with a high level of education participated in the online survey. These characteristics of the sample are typical and should be considered as limiting in the case of studies using online questionnaires [67-69].

The integrated (online access, telephone, and paper mail) National Australian StepUp System for Dementia Research [70] is an interesting solution. In this system, patients with dementia and researchers of the diseases accompanied by cognitive deficits are registered in one of three convenient ways [70]. This allows accelerating the process of collecting data for research hypotheses and developing new approaches to combat dementia [71, 72]. The authors note that the continuous operation of the system went on after the start of the pandemic of a new coronavirus infection [70]. Over the two years of the platform operation, more than 1000 patients, 120 researchers have been registered, and more than 40 studies have been initiated [70].

For clinical trials, there are a number of free services which provide creating electronic individual registration cards, such as REDCap [73] or Ark [74]. The use of specialized services may be limited since access is provided to the organization after the conclusion of an agreement with the copyright holders and not directly to the researcher. However, the service ensures secure personal data storing without third-party access, unlike many open resources, including Google Forms [75]. In the future, research services will be used to create large databases on certain nosologies, diagnostic methods, or treatment. Services are constantly evolving, additional specialized analysis modules are created, for example, building a pedigree [74].

The pandemic of a new coronavirus infection caused an accelerated and forced introduction of digital technologies in all spheres of life, including all stages of research [76, 77]. Since the beginning of the pandemic in 2019, many national and international online monitoring systems have been developed [78]. The challenges for the fast-growing services are their weak integration with each other and the lack of centralized management, a difficulty in interpretation and practical application of data [79]. On the other hand, a limiting factor is the reluctance of patients to use digital questionnaires or remote methods of communication due to uncertainty about confidentiality in their use or unwillingness to become addicted to gadgets [80], which is especially common among older patients.

### Open data

The annual increase in the accumulated data requires the introduction of new guidelines for the management of captured data. One of the most common standards for such work with data is FAIR (findability, accessibility, interoperability, and reusability) [81], which has become a fundamental requirement for open science [82, 83]. In their paper, Suhre et al. [84] emphasize the importance of data exchange for omics research, giving an example of a combination of GWAS and proteomic analysis. The authors consider the prospects for the creation of a database that will accumulate information about the genetic colocalization of genomic information and characteristics of the molecular phenotype of a disease (for example, gene expression and metabolomic characteristics) with clinical trial endpoints.

### Real world data

Real world data in biomedical research refers to data captured from electronic medical records, medical registries, medical insurance companies, non-interventional clinical trials, and other sources in which information was obtained not under experimental conditions [85].

The HealthMap online system (https://www.healthmap.org/ru/) has been operating since 2006, accumulating data on disease outbreaks from open web resources [86]. In 2008, the web-based influenza surveillance system Influenzanet was launched [87, 88]. Limitations in the use of these data are their redundancy (repetitions), heterogeneity (different input formats), inconsistency (violation of the chronology of events). Chatzidimitriou et al. [89] created a database (n=20,463) on clinical cases of chronic lymphocytic leukemia (The ERIC CLL Database) filled with data from more than 90 centers and 31 countries. The authors consider the provision of standardization, integration of retrospective data, and assessment of the quality of input data to be necessary for the successful functioning of the distributed database [89].

### Digital epidemiology as a separate field of knowledge

According to Salathé [90], digital epidemiology has become a separate area of scientific knowledge. Its purpose is to understand the patterns of disease development and the dynamics of the health status of the population, as well as to determine the causes of these patterns in order to find ways to prevent the development of diseases and promote health. The broadest definition of digital epidemiology is epidemiology that uses digital data. Though, the author then specifies that digital epidemiology operates the data that has not been collected with the main purpose of conducting epidemiological studies. Such data can include electronic medical records, information from insurance funds, city, regional, and federal health departments, as well as data from search engines, social networks, and mobile phones [90].

Google Flu Trends (GFT) has become one of the first known digital epidemiology services that uses search queries on acute respiratory symptoms for epidemiological analysis [91, 92]. A serious problem was that the collected data were owned by a private company, and the analysis algorithms used were unavailable even to national healthcare systems [90], and independent testing of the capabilities of this service for epidemiological studies showed a low efficiency in assessing the incidence of infectious diseases [93]. Unofficial Internet sources can be a valuable resource for epidemiological research, but the current trend towards protecting personal data and maintaining privacy is an important limiting factor. Salathé identifies two ways to the solution of this problem [90]:

creation of the monitoring systems by groups of scientists or professional communities, which will be more understandable and transparent for national healthcare, and that will increase the potential for their practical application;

greater involvement of the population in epidemiological studies. The rights to the data generated by individuals belong to the developers of the resource. A representative part of the population should be persuaded to share their personal health data with public health authorities for scientific research, the results of which can benefit society.

**Figure 2.**
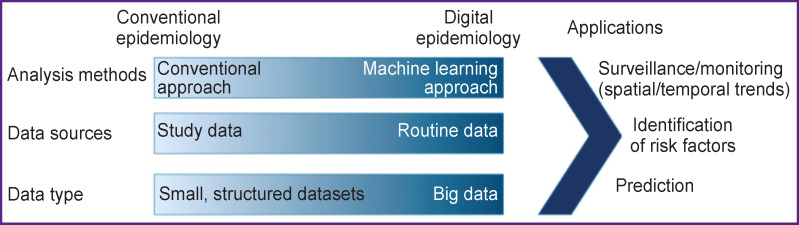
Transition from classical to digital epidemiology [94] Formation of digital epidemiology as a field of knowledge

Roth et al. [94] have shown the formation of digital epidemiology (Figure 2).

According to the authors, machine learning methods based on the data from healthcare systems or social networks (Twitter), which help determine the prognosis for survival and complications, had already been developed by 2018.

It is important to note that the transformation of epidemiology leads to a change in its teaching principles [95]. Werler et al. [96] note that new curricula in epidemiology require the formation of causal thinking and the subsequent formation of a scientific hypothesis. Common mistakes made by young epidemiologists include estimation of one risk factor for one outcome, inaccurate formulation of research questions, and giving greater importance in research to epidemiological and statistical approaches compared to public importance.

## Ethical issues

The development of high-precision medicine technologies entails the need to form new ethical standards [97]. Classical basic ethical principles are respect for patient’s autonomy and privacy [98]. In this case, ethical requirements must ensure that individuals cannot be identified in open data portals for the exchange of scientific data. The ethics of precision public healthcare regulates the interaction between patients who have given voluntary informed consent to their attending doctor for the use of their clinical specimens in precision medicine research and the public decision-making process that drives public health activities. The development of a new hybrid ethical paradigm is possible only with the well-coordinated work of these process participants. Conducting omics studies allows obtaining detailed information about any subject. However, in order to plan disease control measures in a particular area or in a particular population, the following data indicating the demographic characteristics of an individual are important: geographical location, migration history, stay in prison, lifestyle and profession, etc. All these data are personal, they must not be subjected to wide dissemination and increase the risks of discovering the identity of the subject.

In this regard, particular attention is paid to the way of presenting the obtained information. The ethics of precision medicine includes a public health ethic commitment to social justice and an emphasis on professional transparency and the trust built through it. The collected data should be transparent and aimed at improving the existing system and people’s lives, and not stigmatizing social groups with high risk factors or relatively high incidence [97].

The development of electronic systems for capturing and storing data requires careful study of the risks to maintaining the security of the collected data [99]. New requirements for data management and professional confidentiality are emerging [98]. The speed, accuracy, and efficiency of big data processing offer great opportunities for public health, but entail a responsibility to adapt in a society that is committed to privacy, respect for human rights in matters of health, and social justice.

Sharma et al. [100] advocate for the development of legislation to maintain the confidentiality of personal data collected during scientific and clinical research, for auditing and implementation of independent oversight to assess the management of the risks related to the reuse of the data on research subjects. Solving this problem requires new approaches to working with patient data, taking into account an increased activity of scientific communication, creation of open repositories, exchange of primary research data, which is an integral part of large epidemiological studies. However, people are motivated to participate in study by pursuing their own interests, like the reputation of the organization with which they interact. Reuse of data by other organizations carries certain risks, which patients should be informed about before submitting voluntary informed consent to participate in a study.

FAIR-Health is a new paradigm of open science that has been developed in view of the peculiarities of biomedical research [101]. This paradigm is aimed at considering the information and biomaterials collected in research to be a single resource. It is this principle that, according to Holub et al. [101], will help ensure the reproducibility of studies and the subsequent integration of results.

## Conclusion

Modern methods of population-based studies, including both omics technology data and the results of monitoring the conditions and behavior of patients over a long period of time, provide detailed data on subjects. At the moment, a search for methods of standardizing the collected data, their analysis and synthesis for further use is in progress. One of the major challenges to science is the integration of research results not only for rational storage, but also for the creation of dynamic digital models of subjects and processes.

The development of precision medicine technologies underlies the improvement of the quality of life and life expectancy of the population.

## References

[1] Diaz-Beltran L., Cano C., Wall D.P., Esteban F.J. (2013). Systems biology as a comparative approach to understand complex gene expression in neurological diseases.. Behav Sci (Basel).

[2] Wang Q., Lu Q., Zhao H (2015). A review of study designs and statistical methods for genomic epidemiology studies using next generation sequencing.. Front Genet.

[3] Pareek C.S., Smoczynski R., Tretyn A. (2011). Sequencing technologies and genome sequencing.. J Appl Genet.

[4] Hill V., Ruis C., Bajaj S., Pybus O.G., Kraemer M.U.G (2021). Progress and challenges in virus genomic epidemiology.. Trends Parasitol.

[5] Armstrong G.L., MacCannell D.R., Taylor J., Carleton H.A., Neuhaus E.B., Bradbury R.S., Posey J.E., Gwinn M (2019). Pathogen genomics in public health.. N Engl J Med.

[6] Ladner J.T., Grubaugh N.D., Pybus O.G., Andersen K.G (2019). Precision epidemiology for infectious disease control.. Nat Med.

[7] Holmes E.C., Dudas G., Rambaut A., Andersen K.G (2016). The evolution of Ebola virus: insights from the 2013–2016 epidemic.. Nature.

[8] Kulikowski C.A. (2021). Pandemics: historically slow “learning curve” leading to biomedical informatics and vaccine breakthroughs.. Yearb Med Inform.

[9] National Academies of Sciences, Engineering, and Medicine; Division on Earth and Life Studies; Board on Life Sciences; Health and Medicine Division; Board on Health Sciences Policy; (2020). Genomic epidemiology data infrastructure needs for SARS-CoV-2: modernizing pandemic response strategies..

[10] Liu T., Chen Z., Chen W., Chen X., Hosseini M., Yang Z., Li J., Ho D., Turay D., Gheorghe C.P., Jones W., Wang C (2021). A benchmarking study of SARS-CoV-2 whole-genome sequencing protocols using COVID-19 patient samples.. iScience.

[11] Richard M., Knauf S., Lawrence P., Mather A.E., Munster V.J., Müller M.A., Smith D., Kuiken T (2017). Factors determining human-to-human transmissibility of zoonotic pathogens via contact.. Curr Opin Virol.

[12] Gilchrist C.A., Turner S.D., Riley M.F., Petri W.A., Hewlett E.L (2015). Whole-genome sequencing in outbreak analysis.. Clin Microbiol Rev.

[13] Grad Y.H., Lipsitch M (2014). Epidemiologic data and pathogen genome sequences: a powerful synergy for public health.. Genome Biol.

[14] Wohl S., Schaffner S.F., Sabeti P.C (2016). Genomic analysis of viral outbreaks.. Annu Rev Virol.

[15] Maljkovic Berry I., Melendrez M.C., Bishop-Lilly K.A., Rutvisuttinunt W., Pollett S., Talundzic E., Morton L., Jarman R.G. (2020). Next generation sequencing and bioinformatics methodologies for infectious disease research and public health: approaches, applications, and considerations for development of laboratory capacity.. J Infect Dis.

[16] Bandoy D.J.D.R., Weimer B.C (2021). Analysis of SARS-CoV-2 genomic epidemiology reveals disease transmission coupled to variant emergence and allelic variation.. Sci Rep.

[17] Gonzaga-Jauregui C., Lupski J.R., Gibbs R.A. (2012). Human genome sequencing in health and disease.. Annu Rev Med.

[18] Cleeren E., Van der Heyden J., Brand A., Van Oyen H. (2011). Public health in the genomic era: will Public Health Genomics contribute to major changes in the prevention of common diseases?. Arch Public Health.

[19] McGuire A.L., Beskow L.M. (2010). Informed consent in genomics and genetic research.. Annu Rev Genomics Hum Genet.

[20] Picó C., Serra F., Rodríguez A.M., Keijer J., Palou A. (2019). Biomarkers of nutrition and health: new tools for new approaches.. Nutrients.

[21] Fearnley L.G., Inouye M (2016). Metabolomics in epidemiology: from metabolite concentrations to integrative reaction networks.. Int J Epidemiol.

[22] Testa C., Pultrone C., Manners D.N., Schiavina R., Lodi R (2016). Metabolic imaging in prostate cancer: where we are.. Front Oncol.

[23] Luptak I., Sverdlov A.L., Panagia M., Qin F., Pimentel D.R., Croteau D., Siwik D.A., Ingwall J.S., Bachschmid M.M., Balschi J.A., Colucci W.S (2018). Decreased ATP production and myocardial contractile reserve in metabolic heart disease.. J Mol Cell Cardiol.

[24] Metallo C.M., Heiden M.G.V. (2013). Understanding metabolic regulation and its influence on cell physiology.. Mol Cell.

[25] Johnson C.H., Ivanisevic J., Siuzdak G (2016). Metabolomics: beyond biomarkers and towards mechanisms.. Nat Rev Mol Cell Biol.

[26] Bouatra S., Aziat F., Mandal R., Guo A.C., Wilson M.R., Knox C., Bjorndahl T.C., Krishnamurthy R., Saleem F., Liu P., Dame Z.T., Poelzer J., Huynh J., Yallou F.S., Psychogios N., Dong E., Bogumil R., Roehring C., Wishart D.S (2013). The human urine metabolome.. PloS One.

[27] Psychogios N., Hau D.D., Peng J., Guo A.C., Mandal R., Bouatra S., Sinelnikov I., Krishnamurthy R., Eisner R., Gautam B., Young N., Xia J., Knox C., Dong E., Huang P., Hollander Z., Pedersen T.L., Smith S.R., Bamforth F., Greiner R., McManus B., Newman J.W., Goodfriend T., Wishart D.S (2011). The human serum metabolome.. PloS One.

[28] Karu N., Deng L., Slae M., Guo A.C., Sajed T., Huynh H., Wine E., Wishart D.S (2018). A review on human fecal metabolomics: methods, applications and the human fecal metabolome database.. Anal Chim Acta.

[29] Wishart D.S., Lewis M.J., Morrissey J.A., Flegel M.D., Jeroncic K., Xiong Y., Cheng D., Eisner R., Gautam B., Tzur D., Sawhney S., Bamforth F., Greiner R., Li L (2008). The human cerebrospinal fluid metabolome.. J Chromatogr B.

[30] Brown M.V., McDunn J.E., Gunst P.R., Smith E.M., Milburn M.V., Troyer D.A., Lawton K.A (2012). Cancer detection and biopsy classification using concurrent histopathological and metabolomic analysis of core biopsies.. Genome Med.

[31] Robinson O., Keski-Rahkonen P., Chatzi L., Kogevinas M., Nawrot T., Pizzi C., Plusquin M., Richiardi L., Robinot N., Sunyer J., Vermeulen R., Vrijheid M., Vineis P., Scalbert A., Chadeau-Hyam M (2018). Cord blood metabolic signatures of birth weight: a population-based study.. J Proteome Res.

[32] Perng W., Rifas-Shiman S.L., McCulloch S., Chatzi L., Mantzoros C., Hivert M.F., Oken E (2017). Associations of cord blood metabolites with perinatal characteristics, newborn anthropometry, and cord blood hormones in project viva.. Metabolism.

[33] Lau C.E., Siskos A.P., Maitre L., Robinson O., Athersuch T.J., Want E.J., Urquiza J., Casas M., Vafeiadi M., Roumeliotaki T., McEachan R.R.C., Azad R., Haug L.S., Meltzer H.M., Andrusaityte S., Petraviciene I., Grazuleviciene R., Thomsen C., Wright J., Slama R., Chatzi L., Vrijheid M., Keun H.C., Coen M. (2018). Determinants of the urinary and serum metabolome in children from six European populations.. BMC Med.

[34] Petrick L., Edmands W., Schiffman C., Grigoryan H., Perttula K., Yano Y., Dudoit S., Whitehead T., Metayer C., Rappaport S (2017). An untargeted metabolomics method for archived newborn dried blood spots in epidemiologic studies.. Metabolomics.

[35] Roede J.R., Uppal K., Park Y., Lee K., Tran V., Walker D., Strobel F.H., Rhodes S.L., Ritz B., Jones D.P (2013). Serum metabolomics of slow vs. rapid motor progression Parkinson’s disease: a pilot study.. PloS One.

[36] Guasch-Ferré M., Hruby A., Toledo E., Clish C.B., Martínez-González M.A., Salas-Salvadó J., Hu F.B. (2016). Metabolomics in prediabetes and diabetes: a systematic review and meta-analysis.. Diabetes Care.

[37] Wishart D.S., Mandal R., Stanislaus A., Ramirez-Gaona M (2016). Cancer metabolomics and the human metabolome database.. Metabolites.

[38] Frediani J.K., Jones D.P., Tukvadze N., Uppal K., Sanikidze E., Kipiani M., Tran V.T., Hebbar G., Walker D.I., Kempker R.R., Kurani S.S., Colas R.A., Dalli J., Tangpricha V., Serhan C.N., Blumberg H.M., Ziegler T.R (2014). Plasma metabolomics in human pulmonary tuberculosis disease: a pilot study.. PloS One.

[39] Würtz P., Havulinna A.S., Soininen P., Tynkkynen T., Prieto-Merino D., Tillin T., Ghorbani A., Artati A., Wang Q., Tiainen M., Kangas A.J., Kettunen J., Kaikkonen J., Mikkilä V., Jula A., Kähönen M., Lehtimäki T., Lawlor D.A., Gaunt T.R., Hughes A.D., Sattar N., Illig T., Adamski J., Wang T.J., Perola M., Ripatti S., Vasan R.S., Raitakari O.T., Gerszten R.E., Casas J.P., Chaturvedi N., Ala-Korpela M., Salomaa V. (2015). Metabolite profiling and cardiovascular event risk.. Circulation.

[40] Ganna A., Salihovic S., Sundström J., Broeckling C.D., Hedman Å.K., Magnusson P.K.E., Pedersen N.L., Larsson A., Siegbahn A., Zilmer M., Prenni J., Arnlöv J., Lind L., Fall T., Ingelsson E. (2014). Large-scale metabolomic profiling identifies novel biomarkers for incident coronary heart disease.. PLoS Genet.

[41] Rogers A.J., McGeachie M., Baron R.M., Gazourian L., Haspel J.A., Nakahira K., Fredenburgh L.E., Hunninghake G.M., Raby B.A., Matthay M.A., Otero R.M., Fowler V.G., Rivers E.P., Woods C.W., Kingsmore S., Langley R.J., Choi A.M (2014). Metabolomic derangements are associated with mortality in critically ill adult patients.. PloS One.

[42] Playdon M.C., Ziegler R.G., Sampson J.N., Stolzenberg-Solomon R., Thompson H.J., Irwin M.L., Mayne S.T., Hoover R.N., Moore S.C (2017). Nutritional metabolomics and breast cancer risk in a prospective study.. Am J Clin Nutr.

[43] Anfinogenova Y.J., Trubacheva I.A., Serebryakova V.N., Popov S.V (2020). Emerging trends and challenges of population-based cardiology.. Sibirskij zhurnal klinicheskoj i jeksperimental’noj mediciny.

[44] Garg S., Williams N.L., Ip A., Dicker A.P (2018). Clinical integration of digital solutions in health care: an overview of the current landscape of digital technologies in cancer care.. JCO Clin Cancer Inform.

[45] Thyagarajan B., Nelson H.H., Poynter J.N., Prizment A.E., Roesler M.A., Cassidy E., Putnam S., Amos L., Hickle A., Reilly C., Spector L.G., Lazovich D (2020). Field application of digital technologies for health assessment in the 10,000 families study.. Cancer Epidemiol Biomarkers Prev.

[46] Coathup V., Teare H.J., Minari J., Yoshizawa G., Kaye J., Takahashi M.P., Kato K (2016). Using digital technologies to engage with medical research: views of myotonic dystrophy patients in Japan.. BMC Med Ethics.

[47] Briko N.I (2018). Theoretical generalizations in epidemiology: from history to the present.. Epidemiologia i vakcinoprofilaktika.

[48] Jagodzinski A., Johansen C., Koch-Gromus U., Aarabi G., Adam G., Anders S., Augustin M., der Kellen R.B., Beikler T., Behrendt C.A., Betz C.S., Bokemeyer C., Borof K., Briken P., Busch C.J., Büchel C., Brassen S., Debus E.S., Eggers L., Fiehler J., Gallinat J., Gellißen S., Gerloff C., Girdauskas E., Gosau M., Graefen M., Härter M., Harth V., Heidemann C., Heydecke G., Huber T.B., Hussein Y., Kampf M.O, von dem Knesebeck O., Konnopka A., König H.H., Kromer R., Kubisch C., Kühn S., Loges S., Löwe B., Lund G., Meyer C., Nagel L., Nienhaus A., Pantel K., Petersen E., Püschel K., Reichenspurner H., Sauter G., Scherer M., Scherschel K., Schiffner U., Schnabel R.B., Schulz H., Smeets R., Sokalskis V., Spitzer M.S., Terschüren C., Thederan I., Thoma T., Thomalla G., Waschki B., Wegscheider K., Wenzel J.P., Wiese S., Zyriax B.C., Zeller T., Blankenberg S. (2020). Rationale and design of the Hamburg city health study.. Eur J Epidemiol.

[49] Shin Y.K., Koskinen V., Kouvonen A., Kemppainen T., Olakivi A., Wrede S., Kemppainen L (2022). Digital information technology use and transnational healthcare: a population-based study on older Russian-speaking migrants in Finland.. J Immigr Minor Health.

[50] Coppola L., Cianflone A., Grimaldi A.M., Incoronato M., Bevilacqua P., Messina F., Baselice S., Soricelli A., Mirabelli P., Salvatore M (2019). Biobanking in health care: evolution and future directions.. J Transl Med.

[51] Griffon N., Pereira H., Djadi-Prat J., García M.T., Testoni S., Cariou M., Hilbey J., N’Dja A., Navarro G., Gentili N., Nanni O., Raineri M., Chatellier G., Gómez De La Camara A., Lewi M., Sundgren M., Daniel C., Garvey A., Todorovic M., Ammour N. (2020). Performances of a solution to semi-automatically fill eCRF with data from the electronic health record: protocol for a prospective individual participant data meta-analysis.. Stud Health Technol Inform.

[52] Rorie D.A., Flynn R.W.V., Grieve K., Doney A., Mackenzie I., MacDonald T.M., Rogers A. (2017). Electronic case report forms and electronic data capture within clinical trials and pharmacoepidemiology.. Br J Clin Pharmacol.

[53] Mayerhoefer M.E., Materka A., Langs G., Häggström I., Szczypiński P., Gibbs P., Cook G (2020). Introduction to radiomics.. J Nucl Med.

[54] Benelli M., Barucci A., Zoppetti N., Calusi S., Redapi L., Della Gala G., Piffer S., Bernardi L., Fusi F., Pallotta S. (2020). Comprehensive analysis of radiomic datasets by RadAR.. Cancer Res.

[55] Scapicchio C., Gabelloni M., Barucci A., Cioni D., Saba L., Neri E. (2021). A deep look into radiomics.. Radiol Med.

[56] Gagalova K.K., Leon Elizalde M.A., Portales-Casamar E., Görges M. (2020). What you need to know before implementing a clinical research data warehouse: comparative review of integrated data repositories in health care institutions.. JMIR Form Res.

[57] Huser V., Kahn M.G., Brown J.S., Gouripeddi R (2018). Methods for examining data quality in healthcare integrated data repositories.. Pac Symp Biocomput.

[58] Ku W.Y., Nfor O.N., Liu W.H., Tantoh D.M., Hsu S.Y., Wang L., Chou T.Y., Liaw Y.P (2019). Online community collaborative map: a geospatial and data visualization tool for cancer data.. Medicine (Baltimore).

[59] Commons R.J., Thriemer K., Humphreys G., Suay I., Sibley C.H., Guerin P.J., Price R.N (2017). The Vivax Surveyor: online mapping database for Plasmodium vivax clinical trials.. Int J Parasitol Drugs Drug Resist.

[60] Dong E., Du H., Gardner L (2020). An interactive web-based dashboard to track COVID-19 in real time.. Lancet Infect Dis.

[61] Kuzmenkov A.Y., Trushin I.V., Vinogradova A.G., Avramenko A.A., Sukhorukova M.V., Malhotra-Kumar S., Dekhnich A.V., Edelstein M.V., Kozlov R.S (2021). AMRmap: an interactive web platform for analysis of antimicrobial resistance surveillance data in Russia.. Front Microbiol.

[62] Liu Y., Elsworth B., Erola P., Haberland V., Hemani G., Lyon M., Zheng J., Lloyd O., Vabistsevits M., Gaunt T.R (2021). EpiGraphDB: a database and data mining platform for health data science.. Bioinformatics.

[63] Hemani G., Zheng J., Elsworth B., Wade K.H., Haberland V., Baird D., Laurin C., Burgess S., Bowden J., Langdon R., Tan V.Y., Yarmolinsky J., Shihab H.A., Timpson N.J., Evans D.M., Relton C., Martin R.M., Davey Smith G., Gaunt T.R., Haycock P.C. (2018). The MR-Base platform supports systematic causal inference across the human phenome.. eLife.

[64] Slenter D.N., Kutmon M., Hanspers K., Riutta A., Windsor J., Nunes N., Mélius J., Cirillo E., Coort S.L., Digles D., Ehrhart F., Giesbertz P., Kalafati M., Martens M., Miller R., Nishida K., Rieswijk L., Waagmeester A., Eijssen L.M.T., Evelo C.T., Pico A.R., Willighagen E.L (2018). WikiPathways: a multifaceted pathway database bridging metabolomics to other omics research.. Nucleic Acids Res.

[65] Szklarczyk D., Morris J.H., Cook H., Kuhn M., Wyder S., Simonovic M., Santos A., Doncheva N.T., Roth A., Bork P., Jensen L.J., von Mering C. (2017). The STRING database in 2017: quality-controlled protein-protein association networks, made broadly accessible.. Nucleic Acids Res.

[66] Toom K., Raidvee A., Braschinsky M (2020). The applicability of web-based solutions in headache epidemiology research.. J Headache Pain.

[67] Egger M.J., Lukacz E.S., Newhouse M., Wang J., Nygaard I (2013). Web versus paper-based completion of the epidemiology of prolapse and incontinence questionnaire.. Female Pelvic Med Reconstr Surg.

[68] Tassiopoulos K., Roberts-Toler C., Fichtenbaum C.J., Koletar S.L (2020). Web-based data collection for older adults living with HIV in a clinical research setting: pilot observational study.. J Med Internet Res.

[69] Braekman E., Charafeddine R., Demarest S., Drieskens S., Berete F., Gisle L., Van der Heyden J., Van Hal G. (2020). Comparing web-based versus face-to-face and paper-and-pencil questionnaire data collected through two Belgian health surveys.. Int J Public Health.

[70] Jeon Y.H., Shin M., Smith A., Beattie E., Brodaty H., Frost D., Hobbs A., Kotting P., Petrie G., Rossor M., Thompson J., Vickers J., Waters D (2021). Early implementation and evaluation of StepUp for Dementia Research: an Australia-wide dementia research participation and public engagement platform.. Int J Environ Res Public Health.

[71] Langbaum J.B., Karlawish J., Roberts J.S., Wood E.M., Bradbury A., High N., Walsh T.L., Gordon D., Aggarwal R., Davis P., Stowell C., Trisko L., Langlois C.M., Reiman E.M., Tariot P.N (2019). GeneMatch: a novel recruitment registry using at-home APOE genotyping to enhance referrals to Alzheimer’s prevention studies.. Alzheimers Dement J Alzheimers Assoc.

[72] Weiner M.W., Nosheny R., Camacho M., Truran-Sacrey D., Mackin R.S., Flenniken D., Ulbricht A., Insel P., Finley S., Fockler J., Veitch D (2018). The Brain Health Registry: an internet-based platform for recruitment, assessment, and longitudinal monitoring of participants for neuroscience studies.. Alzheimers Dement J Alzheimers Assoc.

[73] Harris P.A., Taylor R., Minor B.L., Elliott V., Fernandez M., O’Neal L., McLeod L., Delacqua G., Delacqua F., Kirby J., Duda S.N (2019). REDCap Consortium. The REDCap consortium: building an international community of software platform partners.. J Biomed Inform.

[74] Ranaweera T., Makalic E., Hopper J.L., Bickerstaffe A (2018). An open-source, integrated pedigree data management and visualization tool for genetic epidemiology.. Int J Epidemiol.

[75] Djenno M., Insua G.M., Pho A (2015). From paper to pixels: using Google Forms for collaboration and assessment.. Libr Hi Tech News.

[76] Wang X (2021). Establishment of an internet-based epidemiological survey data collection customized system model.. Front Public Health.

[77] Golinelli D., Boetto E., Carullo G., Nuzzolese A.G., Landini M.P., Fantini M.P (2020). Adoption of digital technologies in health care during the COVID-19 pandemic: systematic review of early scientific literature.. J Med Internet Res.

[78] Budd J., Miller B.S., Manning E.M., Lampos V., Zhuang M., Edelstein M., Rees G., Emery V.C., Stevens M.M., Keegan N., Short M.J., Pillay D., Manley E., Cox I.J., Heymann D., Johnson A.M., McKendry R.A (2020). Digital technologies in the public-health response to COVID-19.. Nat Med.

[79] He Z., Zhang C.J.P., Huang J., Zhai J., Zhou S., Chiu J.W., Sheng J., Tsang W., Akinwunmi B.O., Ming W.K. (2020). A new era of epidemiology: digital epidemiology for investigating the COVID-19 outbreak in China.. J Med Internet Res.

[80] Horst B.R., Sixsmith A., Simeonov D., Mihailidis A (2021). Demographic and psychographic factors of social isolation during the COVID-19 pandemic: the importance of technology confidence.. Front Public Health.

[81] Wilkinson M.D., Dumontier M., Jan Aalbersberg I., Appleton G., Axton M., Baak A., Blomberg N., Boiten J.W., da Silva Santos L.B., Bourne P.E., Bouwman J., Brookes A.J., Clark T., Crosas M., Dillo I., Dumon O., Edmunds S., Evelo C.T., Finkers R., Gonzalez-Beltran A., Gray A.J.G., Groth P., Goble C., Grethe J.S., Heringa J., ‘t Hoen P.A.C., Hooft R., Kuhn T., Kok R., Kok J., Lusher S.J., Martone M.E., Mons A., Packer A.L., Persson B., Rocca-Serra P., Roos M., van Schaik R., Sansone S.A., Schultes E., Sengstag T., Slater T., Strawn G., Swertz M.A., Thompson M., van der Lei J., van Mulligen E., Velterop J., Waagmeester A., Wittenburg P., Wolstencroft K., Zhao J., Mons B. (2019). Addendum: the FAIR Guiding Principles for scientific data management and stewardship.. Sci Data.

[82] Munafò M.R., Hollands G.J., Marteau T.M. (2018). Open science prevents mindless science.. BMJ.

[83] Sanjana N.E (2021). Voices of the new generation: open science is good for science (and for you).. Nat Rev Mol Cell Biol.

[84] Suhre K., McCarthy M.I., Schwenk J.M (2021). Genetics meets proteomics: perspectives for large population-based studies.. Nat Rev Genet.

[85] Bian J., Lyu T., Loiacono A., Viramontes T.M., Lipori G., Guo Y., Wu Y., Prosperi M., George T.J., Harle C.A., Shenkman E.A., Hogan W (2020). Assessing the practice of data quality evaluation in a national clinical data research network through a systematic scoping review in the era of real-world data.. J Am Med Inform Assoc.

[86] Brownstein J.S., Freifeld C.C., Reis B.Y., Mandl K.D (2008). Surveillance Sans Frontières: internet-based emerging infectious disease intelligence and the HealthMap project.. PLoS Med.

[87] Land-Zandstra A.M., van Beusekom M., Koppeschaar C., van den Broek J. (2016). Motivation and learning impact of Dutch flu-trackers.. J Sci Commun.

[88] Paolotti D., Carnahan A., Colizza V., Eames K., Edmunds J., Gomes G., Koppeschaar C., Rehn M., Smallenburg R., Turbelin C., Van Noort S., Vespignani A. (2014). Web-based participatory surveillance of infectious diseases: the Influenzanet participatory surveillance experience.. Clin Microbiol Infect.

[89] Chatzidimitriou A., Minga E., Chatzikonstantinou T., Moreno C., Stamatopoulos K., Ghia P (2020). Challenges and solutions for collecting and analyzing real world data: the Eric CLL database as an illustrative example.. Hemasphere.

[90] Salathé M. (2018). Digital epidemiology: what is it, and where is it going?. Life Sci Soc Policy.

[91] Ginsberg J., Mohebbi M.H., Patel R.S., Brammer L., Smolinski M.S., Brilliant L. (2009). Detecting influenza epidemics using search engine query data.. Nature.

[92] Lippi G., Mattiuzzi C., Cervellin G (2019). Is digital epidemiology the future of clinical epidemiology?. J Epidemiol Glob Health.

[93] Cervellin G., Comelli I., Lippi G (2017). Is Google Trends a reliable tool for digital epidemiology? Insights from different clinical settings.. J Epidemiol Glob Health.

[94] Roth J.A., Battegay M., Juchler F., Vogt J.E., Widmer A.F (2018). Introduction to machine learning in digital healthcare epidemiology.. Infect Control Hosp Epidemiol.

[95] Frérot M., Lefebvre A., Aho S., Callier P., Astruc K., Aho Glélé L.S. (2018). What is epidemiology? Changing definitions of epidemiology 1978–2017.. PLoS One.

[96] Werler M.M., Stuver S.O., Healey M.A., LaMorte W.W (2019). The future of teaching epidemiology.. Am J Epidemiol.

[97] Vallury K.D., Baird B., Miller E., Ward P (2021). Going viral: researching safely on social media.. J Med Internet Res.

[98] Juengst E.T., Van Rie A. (2020). Transparency, trust, and community welfare: towards a precision public health ethics framework for the genomics era.. Genome Med.

[99] Solomon D.H., Rudin R.S (2020). Digital health technologies: opportunities and challenges in rheumatology.. Nat Rev Rheumatol.

[100] Sharma A., Nilsen T.B., Czerwinska K.P., Onitiu D., Brenna L., Johansen D., Johansen H.D (2021). Up-to-the-minute privacy policies via gossips in participatory epidemiological studies.. Front Big Data.

[101] Holub P., Kohlmayer F., Prasser F., Mayrhofer M.T., Schlünder I., Martin G.M., Casati S., Koumakis L., Wutte A., Kozera Ł., Strapagiel D., Anton G., Zanetti G., Sezerman O.U., Mendy M., Valík D., Lavitrano M., Dagher G., Zatloukal K., van Ommen G.B., Litton J.E. (2018). Enhancing reuse of data and biological material in medical research: from FAIR to FAIR-Health.. Biopreserv Biobank.

